# Petri graph neural networks advance learning higher order multimodal complex interactions in graph structured data

**DOI:** 10.1038/s41598-025-01856-9

**Published:** 2025-05-20

**Authors:** Alma Ademovic Tahirovic, David Angeli, Adnan Tahirovic, Goran Strbac

**Affiliations:** 1 Department of Electrical and Electronic Engineering, Imperial College London, SW7 2AZ London, UK; 2 Intelligent Systems Hub, 71000 Sarajevo, Bosnia and Herzegovina; 3 Department of Information Engineering, University of Florence, 50139 Florence, Italy; 4 Department of Automatic Control and Electronics, Faculty of Electrical Engineering, University of Sarajevo, 71000 Sarajevo, Bosnia and Herzegovina

**Keywords:** Heterogeneous network flow, Higher-order complex networks, Hypergraphs, Multilayer networks, Multimodal data, Petri nets, Information theory and computation, Complex networks

## Abstract

Graphs are widely used to model interconnected systems, offering powerful tools for data representation and problem-solving. However, their reliance on pairwise, single-type, and static connections limits their expressive capacity. Recent developments extend this foundation through higher-order structures, such as hypergraphs, multilayer, and temporal networks, which better capture complex real-world interactions. Many real-world systems, ranging from brain connectivity and genetic pathways to socio-economic networks, exhibit multimodal and higher-order dependencies that traditional networks fail to represent. This paper introduces a novel generalisation of message passing into learning-based function approximation, namely multimodal *heterogeneous network flow*, which models information propagation across different semantic domains under conservation constraints. This framework is defined via Petri nets, which extend hypergraphs to support concurrent, multimodal flow and richer structural representation. Building on this foundation, we present the *Petri Graph Neural Network* (PGNN), a new class of graph neural networks capable of learning over higher-order, multimodal structures. PGNN generalises message passing by incorporating flow conversion and concurrency, leading to enhanced expressive power, interpretability, and computational efficiency. The work opens new directions in learning over complex structures, transcending transformer-based and traditional hypergraph-based algorithms. We validate results through theoretical analysis and real-world experiments, while demonstrating superior performance in, e.g., stock market prediction.

## Introduction

*Graphs* correspond to an extensively utilised concept in the study of interconnected subsystems, formalising physical or abstract connections^1,2^, decoding structure in data^3–7^, or providing tools to transform and solve problems in new and insightful ways^8–10^. Due to pairwise relationships over single type and static topologies, however, graphs come with certain limitations in providing sufficient expressive power^11–14^. Recent generalisations draw attention to higher-order complex structures, such as: *hypergraphs*, allowing any number of nodes to participate in a connection^11,15^, *multilayer networks*, encoding multiple types of nodes and connections^16–19^, and *temporal networks*, whose connections change over time^20^. Interaction is, furthermore, often described as a form of evolution process, which under steady state conditions conforms to a *network flow*^8,9^ (i.e., random walk, belief propagation, or message passing^21,22^). Our recent work provides a generalisation of network flow with respect to higher-order complex structures, in the form of multimodal *heterogeneous network flow*^23^. Unlike traditional network flow, the framework allows flow derivation across different semantic domains (i.e., it encodes a type of flow conversion, satisfying conservation constraints; informal rationale can be found in a number of interacting subsystems, e.g., chemical processes, energy networks, logistics, finance, or any other form of conversion process relying on the laws of conservation). The framework is defined with respect to a *Petri net*^24,25^, a generalised hypergraph maintaining a form of multilayer concurrency. Unlike a hypergraph, the object encodes a model of *multimodal information flow*^23,24,26–29^, focusing on concurrent event systems and topological representation of the underlying relationship structure, offering an added level of expressive power.

**Fig. 1:**
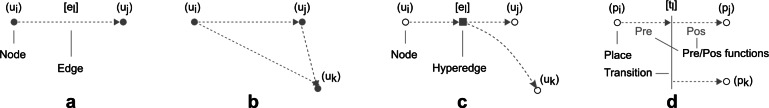
Illustrative example of a graph, hypergraph, and Petri net. **(a)** *Graph*, determined by edge $e_{l} = \{ u_{i},u_{j} \} \in E$ (lines), and incident nodes $u_{i}, u_{j} \in V$ (dots). (**b**) “Three-body” interaction expressed as a graph (i.e., three pairwise interactions). (**c**) *Hypergraph*, determined by hyperedge $e_{l} = \{ \{ u_{i} \}, \{ u_{j},u_{k} \} \} \in E_{H}$ (rectangles), and incident nodes $u_{i}, u_{j}, u_{k} \in V$ (circles). Three-body interaction expressed as a hypergraph. (**d**) *Petri net*, determined by transition $t_{l} = \{ \{ p_{i} \}, \{ p_{j},p_{k} \} \} \in E_{H}$ (bars), incident place nodes $p_{i}, p_{j}, p_{k} \in V$ (circles), and *Pre*, *Pos* functions. Three-body interaction expressed as a Petri net.

This is important as recent research has demonstrated that most real-world systems, e.g.: functional and structural brain networks^30–33^, protein interaction networks^34^, metabolic and genetic systems^35^, social coordination^36^, or group formation networks^37^, to name a few, all expand beyond traditional graph-based intuition, while exhibiting higher-order complex network features^11–13,38,39^. Traditional graph methods do not capture higher-order multimodal node relationships, due to limited scope of expression (e.g., a triangle of three nodes $u_{i}, u_{j}, u_{k}$, expressed as three links $\{u_{i}, u_{j}\}, \{u_{i}, u_{k}\}, \{u_{j}, u_{k}\}$ in a graph, does not capture the difference between a three-body interaction (a hypergraph) (see, Fig. 1c), and coexistence of three pairwise interactions (a hypergraph special case) (see, Fig. 1a,b), two entirely different cases^40^; furthermore, a hypergraph does not capture the multilayer concurrent structure encoded in a Petri net (see, Fig. 1c,d), given no notion of multimodal node interaction^11,16,17^, flow conversion (i.e., *Pre*, *Pos* relationship)^23–25^, or parallel interplay between different semantic domains^23^). This is important in a number of applications involving node interaction, including in learning-based function approximation, expressed by information propagation mechanisms over graph-like structures. Such concepts include *Graph Neural Networks (GNN)*^7,41^, an extension of a recursive neural network, building on the notion of message passing over graph-like objects^42,43^. To that end, this paper introduces a novel paradigm, i.e., the *Petri Graph Neural Network*, an extension of the traditional graph neural network, generalising message passing with respect to higher-order complex structures, while offering more expressive power, interpretability, and computational efficiency. Properties are corroborated by theoretical proof and empirical evidence, while identifying higher-order multimodal complex interaction in, e.g., stock market price prediction.

The remainder of this paper is organised as follows. In “Preliminaries” an overview of mathematical background concepts is presented. The “Graph neural networks” section sets out the GNN methodology and limitations. The proposed PGNN framework is introduced under “Proposed framework”, while illustrative and real-world examples are presented in “Examples”. Concluding remarks and future work are summarised in “Conclusions”.

## Results

### Preliminaries

To aid further understanding, a brief introduction of background concepts is presented first, including basic terminology and notation. The formal notions of: graphs, hypergraphs, multilayer networks, Petri nets, network flows, and heterogeneous network flows, are introduced individually, by recalling some relevant definitions.

#### Graphs

A *graph* $G = (V, E)$^44^ (Fig. 1a,b), is a collection of *n* elements $u \in V$ called *nodes* (vertices), and *m* *edges* connecting pairs of nodes $\{u,v\} \in E \subseteq [V]^{2}$, such that $[V]^{2}$ denotes all two-element subsets of *V*. An *adjacency matrix* $A_{m} \in {\mathbb {R}}^{(n \times n)}$ encodes all vertex pairs of *E*, while an *incidence matrix* $A_{t} \in {\mathbb {R}}^{(n \times m)}$ encodes all incident vertices and edges. The *neighbourhood* of vertex *u* is denoted as ${\mathcal {N}}(u) = \{ v \in V \mid \{u,v\} \in E \}$. Where multiple edges between adjacent nodes are allowed, such that *E* is no longer a subset of $[V]^{2}$, but just another finite set $V \cap E = \varnothing$, with $\mu :E \rightarrow [V]^{2}$, object $G=(V,E,\mu )$ becomes a *multigraph*. The graph can be weighted $\omega : E \rightarrow {\mathbb {R}}^{+}$ (${\mathbb {R}}^{+} = \{ x \in {\mathbb {R}} \mid x>0 \}$) or unweighted, where $\omega$ denotes the *weighting coefficient*. The graph is assumed *connected*, or otherwise decomposed into the union of its connected components.

#### Hypergraphs

As graphs explicitly encode *pairwise* node interactions, they are successful in capturing properties of systems expressed in two-body terms^45^ (Fig. 1a,b). Their limits, however, emerge in models of *higher-order interactions*, e.g., a triangle of three nodes $u_{i}, u_{j}, u_{k}$, expressed as three links $\{u_{i}, u_{j}\}, \{u_{i}, u_{k}\}, \{u_{j}, u_{k}\}$ in a graph, is not able to capture the difference between a three-body interaction and the coexistence of three pairwise interactions, two entirely different cases^40^ (Fig. 1b,c). A *hypergraph* $H = (V,E_{H})$^15^, on the other hand, allows any number of nodes to participate in an edge, i.e., *hyperedge*, of edge set $E_{H} \subseteq {\mathcal {P}}(V)$, where ${\mathcal {P}}(V)$ denotes a power set of *V*, i.e., the set of all subsets of *V*. Analogously, a *multi-hypergraph* $H = (V,E_{H},\mu _{H})$, is an object such that $\mu _{H}:E_{H} \rightarrow {\mathcal {P}}(V)$. It is worth adding that, while hypergraphs are more expressive than graphs and other relational objects such as simplicial complexes, they are harder to analyse, with many concepts useful for graphs yet to be extended to the hypergraph setting^11^.

**Fig. 2:**
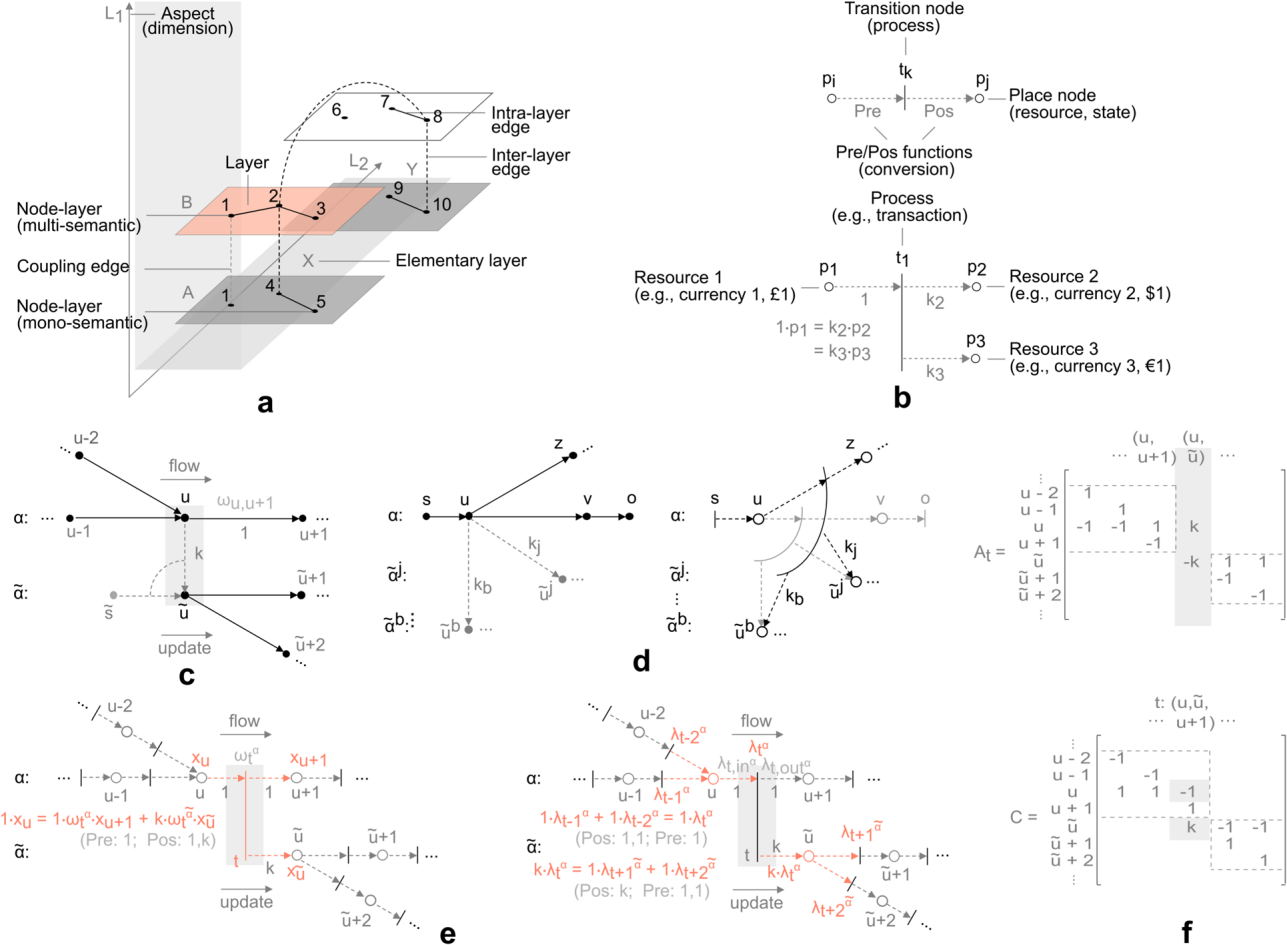
Simplified illustration of multilayer network, Petri net, heterogeneous flow network, and network transformation^23^ (see, “Multilayer networks”, “Petri nets”, and “Heterogeneous network flow”, for details and notation). (**a**) Multilayer network $$M = (V_{M},E_{M},V,L_{M})$$, consisting of a vertex set $$V = \{1,\ldots ,10\}$$, and a layer sequence $$L_{M}$$, comprising sets of elementary layers $$L_{1} = \{A,B\}$$, $$L_{2} = \{X,Y\}$$, with two aspects $$d = 2$$. The network consists of four layers, $$\alpha ^{i} \equiv (\alpha _{1}^{i}, \alpha _{2}^{i}), i \in \{1,\ldots ,4\}: (A,X), (A,Y), (B,X), (B,Y)$$, a node-layer set $$V_{M} = \{(1,A,X), (4,A,X), (5,A,X), (9,A,Y), (10,A,Y), (1,B,X),\ldots , (8,B,Y)\}$$, and an edge set $$E_{M}$$, comprising intra-layer edge set $$E_{A}$$ (solid lines), inter-layer edge set $$E_{C}$$ (dashed lines), and coupling edge set $$E_{{\bar{C}}}$$ (dashed grey lines, interpretable as a *split node*^9^, i.e., one node residing in multiple layers, e.g., node-layer $$(1,\alpha ^{i})$$, $$i \in \{1,3\}$$; the distinction with respect to inter-layer edges is important, as this object is interpretable as a *node* in a *bipartite* structure, e.g., transition node, rather than an arc - for reference, see next panel). (**b**) Petri net, with place nodes $$p_{i}, p_{j} \in P$$ (circles) interpretable as resources or system states, and transition nodes $$t_{k} \in T$$ (bars) interpretable as processes, along with *Pre* and *Pos* functions (arcs), interpretable as conversion ratios or weights (top figure) (e.g., 1 unit of resource $$p_{1}$$, produces $$k_{2}$$ units of resource $$p_{2}$$, and $$k_{3}$$ units of resource $$p_{3}$$, through process $$t_{1}$$ - bottom figure). (**c**) Heterogeneous flow network $$H_{f}$$ and node-layer *u*, residing in layers $$\alpha$$, $${\tilde{\alpha }}$$, and forming a split node link $$(u,{\tilde{u}})$$, acting as exogenous arc with respect to sink layer $${\tilde{\alpha }}$$ (mapped to single-source $${\tilde{s}}$$ as flow circulation). (**d**) Transformation of heterogeneous flow network (left-hand side), to line-graph Petri net equivalent (right-hand side), where arcs (lines) match transition nodes (bars), while nodes (dots) match places (circles) with corresponding end node transitions. Single-source and single-sink arcs, (*s*, *u*) and (*v*, *o*), are mapped as transition nodes, corresponding to sources and sinks of exogenous flow (for further details on the transformation, e.g., bidirectional, positive, negative, transpose arcs or flow conversion, please refer to^23^). (**e**) Breakdown of Petri net equivalent of network in panel (c), with respective relations of: mass conservation (i.e., semantic conversion, left panel; e.g., in financial networks input and output cash flows need to add up, regardless of currency, e.g., $$w_{1,t}^{\alpha _{1}} \cdot$$ £1 = $$w_{2,t}^{\alpha _{2}} \cdot$$ $ 1 $$+$$ $$w_{3,t}^{\alpha _{3}} \cdot$$ euro 1, $$w_{u,t} = k_{u} \circ \omega _{t}^{\alpha _{u}}$$; note that *Pre* and *Pos* functions (e.g., $$k_{u}$$), attached to links, encode conversion, while weights $$\omega _{t}^{\alpha }$$, attached to transitions *t* and layers $$\alpha$$, encode flow rates, which together with potentials *x* determine flows, e.g., $$\lambda _{t}^{\alpha } = \omega _{t}^{\alpha } \circ x_{u}$$); and flow balance (right panel, i.e., classic form of identity, e.g., $$\lambda _{t,in}^{\alpha } = \lambda _{t,out}^{\alpha } = \lambda _{t}^{\alpha }$$; note that *Pre* and *Pos* functions encode conversion, hence flow to a new semantic layer $${\tilde{\alpha }}$$, i.e., $$\lambda _{t}^{{\tilde{\alpha }}}$$ (note, always exogenous), equals $$\lambda _{t}^{{\tilde{\alpha }}} = k \circ \lambda _{t}^{\alpha }$$, derived from concurrent flow $$\lambda _{t}^{\alpha }$$ in layer $$\alpha$$). (**f**) Illustration of *incidence matrix* correspondence, where $$A_{t}$$ refers to graph incidence matrix of network in panel (c) (unweighted, without loss of generality, except for link $$(t,{\tilde{u}})$$), while *C* corresponds to Petri net incidence (coupling) matrix of network in panel (e) (note that off-diagonal block elements in *C* correspond to a form of “inter-layer” interaction, encoding concurrency).

#### Multilayer networks

A *multilayer network* $M = (V_{M},E_{M},V,L_{M})$^16,17^ (Fig. 2a), is a generalised form of a graph, consisting of *b* *layers* $\alpha ^{i}$, $i \in \{ 1,\ldots ,b \}$, each an array $(\alpha _{1}^{i},\ldots ,\alpha _{d}^{i})$ of *elementary layers* $\alpha _{a}^{i}$, corresponding to *d* aspects $a \in \{ 1,\ldots ,d \}$ (which informally can be thought of as dimensions). The *layer sequence* $L_{M} = \{ L_{a} \}_{a=1}^{d}$, consists of sets of elementary layers $L_{a} = \{ \alpha \mid \exists i \in \{ 1,\ldots ,b \}, \alpha _{a}^{i} = \alpha \}$, where nodes and layers form a multilayer node set $V_{M} \subseteq V \times L_{1} \times \ldots \times L_{d}$, comprising of *node-layers* $(u,\alpha ^{i}) \equiv (u, \alpha _{1}^{i},\ldots , \alpha _{d}^{i})$ (i.e., *u* denotes a node $u \in V$ existing on elementary layers $\alpha _{a}^{i}$). The multilayer edge set $E_{M} \subseteq [V_{M}]^{2}$ is a two-element subsets of $V_{M}$, where *intra-layer edge set* $E_{L} = \{ \{ (u,\alpha ^{i}),(v,\alpha ^{j}) \} \in E_{M} \mid \alpha ^{i} = \alpha ^{j} \}$, and *inter-layer edge set* $E_{C} = E_{M} \setminus E_{L}$. A coupling edge set is a subset $E_{{\bar{C}}} \subseteq E_{C}$, $E_{{\bar{C}}} = \{ \{ (u,\alpha ^{i}),(v,\alpha ^{j}) \} \in E_{C} \mid u = v \}$. A multilayer arc set is $A_{M} \subseteq V_{M} \times V_{M}$. If the object is weighted, $\omega _{M}: E_{M} \rightarrow {\mathbb {R}}^{+}$. The traditional notion of a graph is a *single-layer network* or *monoplex*.

#### Petri nets

A *Petri net* $P_{N} = (P,T,Pre,Pos)$^24,25^ (Fig. 1d), is an object consisting of |*P*| *place* nodes $p_{i} \in P$, and |*T*| *transition* nodes $t_{j} \in T$, with $Pre: P \times T \rightarrow {\mathbb {R}}_{0}^{+}$ an incidence function specifying weights from places to transitions, and $Pos: P \times T \rightarrow {\mathbb {R}}_{0}^{+}$ an incidence function specifying weights from transitions to places. In a physical sense, place nodes can informally be understood as resources or system states, while transition nodes can be understood as processes (Fig. 2b). The Petri net *incidence matrix* is derived as $C = Pos-Pre$. A *P-invariant* is a vector of the form $x \in {\mathbb {R}}_{0}^{+^{n}}$, where $x^{T} C = 0$ with support set $\Vert x \Vert = \{ p_{i} \in P: x_{i}>0 \}$. A *T-invariant* is a vector of the form $y \in {\mathbb {R}}_{0}^{+^{m}}$, where $C y = 0$ with support set $\Vert y \Vert = \{ t_{j} \in T: y_{j}>0 \}$. The two relations encode fundamental laws of *mass conservation* (*P*-invariant), and *flow balance* (*T*-invariant)^46^. The Petri net is a model of information flow, with specific focus on *concurrent event* systems.

#### Flow networks

A *flow network* $N_{f}=(V,A,c,s,o)$^8,9,44^, is a digraph consisting of directed edges (ordered node-pairs) or arcs $(u,v) \in A \subseteq V \times V$, an *arc capacity function* $c:A \rightarrow {\mathbb {R}}^{+}$, a set of *source* nodes, $S \subseteq V$, and *sink* (output) nodes, $O \subseteq V$ ($S \cap O = \emptyset$). From a physical point of view, the subsets define the sets of points where flow enters or leaves a network, in form of *exogenous flow*. Exogenous flow can be transformed to a *flow circulation*, with auxiliary single-source $s \in V$ and single-sink $o \in V$, acting as the *environment*, where *s* is incident with all vertices $S = \{ u \mid (s,u) \in A \}$, such that $(v,s) \notin A, \forall v \in V$, while *o* is incident with all vertices $O = \{ v \mid (v,o) \in A \}$, such that $(o,u) \notin A, \forall u \in V$. Arc capacity from and to the environment is assumed infinite. The *direction of flow* corresponds to the direction of an arc (providing physical meaning), hence bidirectional flow is represented by two oppositely facing arcs. A *network flow* is a function $f:A \rightarrow {\mathbb {R}}_{0}^{+}$ defined with respect to the topology of a flow network $N_{f}$. It satisfies conditions of: (i) flow capacity ($0 \le f \le c$); (ii) conservation of flow ($A_{t}f = 0$); and (iii) direction of flow ($f(u,v) \cdot f(v,u) = 0, \forall (u,v) \in \{ (u,v) \in A \mid (v,u) \in A \}$).

#### Heterogeneous network flow

Multimodal network flow is a flow function introduced with respect to a: *heterogeneous flow network* (HFN) $H_{f}$, the multilayer equivalent of a Petri net $P_{N}$, and *heterogeneous network flow*, the higher-order multilayer flow function allowing derivation of multimodal algebraic flow^23^ (Fig. 2c). $T_{f}: H_{f} \rightarrow P_{N}$ is an *invertible HFN-Petri net transformation* (Fig. 2d) (for detailed formal introduction see^23^,), which flattens the layered relationship structure of $H_{f}$ to a form of bipartite graph or Petri net $P_{N}$, at the expense of losing explicit semantics (simplifying multimodal flow computation). Given reversible correspondence, however, interpretation is always preserved and outputs can uniquely be transformed from one mathematical object to the other. Under transformation, the HFN corresponds to a hypergraph, with the addition of a multilayer network structure.

Heterogeneous network flow is a function encoding: *transfer*, a classic form of intra-layer network flow, and *coupling*, a form of inter-layer flow establishing exogenous relationship between layers in the form of transformation. Unlike traditional network flow, the function satisfies both flow and mass conservation (Fig. 2e), due to Petri net correspondence. Formally, given a heterogeneous flow network $H_{f}$ and intra-layer arc set $A_{L} \subseteq A_{M}$, a heterogeneous network flow is a flow function $f_{h}: A_{L} \rightarrow {\mathbb {R}}_{0}^{+}$, such that the vector $\lambda$ defined as

1 $$\begin{aligned} [\lambda (t)]_{t \in T} = [f_{h}({\tilde{x}},{\tilde{y}})]_{({\tilde{x}},{\tilde{y}}) \in A_{L}}, \end{aligned}$$

where edges $({\tilde{x}},{\tilde{y}}) \in A_{L}$ are taken in the same order as the corresponding transitions $t \in T$, fulfils

2 $$\begin{aligned} C \lambda = 0, \end{aligned}$$

where *C* is an incidence matrix (Fig. 2f), and $\lambda \in {\mathbb {R}}_{0}^{+^{m}}$ a stationary firing rate of a Petri net $P_{N}$^23^.

### Related work

#### Graph neural networks - Framework & limitations

GNNs come in different forms and vary by application, i.e.: *Graph Convolution Networks (GCN)* extend convolutions to graphs^47,48^; *Graph Attention Networks (GAN)*^49–51^ assign weights to nodes of the same neighbourhood to account for different levels of influence over targets; *Graph SAmple and AggreGatE Networks* (GraphSAGE)^52^ provide inductive representation learning over large graphs, efficiently generalising to unseen data; while *Graph Isomorphism Networks* (GIN)^53^ generalise observations with respect to aggregations in the form of aggregate (pass) and combine (update) functions. The different GNN classes, however, ultimately all correspond to a common GNN concept^54^, namely the *Message Passing Neural Network (MPNN)*^42,43^, implementing information propagation mechanisms and learning graphical models in the embedding feature space directly, while optimising the aggregation function at the same time. This is justified by the property that embeddings represent a sufficient statistic corresponding to the original latent variable distribution function^55^. To account for some forms of higher-order network structures, further extensions were later introduced in form of: *Heterogeneous Graph Neural Networks (HGNN)*^56,57^, *Heterogeneous Graph Attention Networks (HGAN)*^58,59^, *Message Passing Heterogeneous Neural Networks (MPHNN)*^60^, and *Hypergraph Neural Networks (HYGNN)*^61,62^, where the latter incorporates the concept of learning over hypergraphs^63^. Other concepts include *Multiset Hypergraph Neural Networks (AllSet)*^64^, *Unified Framework for Graph and Hypergraph Neural Networks (UniGNN)*^65^, or *Equivariant Hypergraph Diffusion Neural Networks (EDHNN)*^66^, incorporating a form of hypergraph diffusion model, effectively a special case of a continuous timed Petri net^26–29^. A variant of higher-order message passing was also introduced in form of, e.g., *Hypergraph Message Passing (HYPER-MP)*^67,68^, as well as transformers on graphs, e.g., *Tokenized Graph Transformers (TOKEN-GT)*^69^, exhibiting the highest level of expressive power among current state-of-the-art methods, as powerful as the *k-Weisfeiler-Lehman *(*k-WL*) graph isomorphism test^67–72^ (which effectively explores structure). However, these frameworks still disregard directionality in the definition of a relationship (i.e., they encode symmetry with respect to semantic conversion; e.g., $2H_{2}+O_{2} \rightarrow 2H_{2}O$, but also all other possible combinations thereof, such as $2H_{2}+2H_{2}O \rightarrow O_{2}$, which is clearly inconsistent with the physical meaning of the process), and cannot express simultaneous n-body and multilayer node interaction (i.e., multi-body relationships and semantic conversion; this is, furthermore, corroborated by the fact that traditional higher-order complex structures are *expressed as independent objects* by definition^11–14^, see “Preliminaries”, separating the different types of higher-order node dependencies^11^; a detailed overview is presented in “Proposed framework” and “Examples”), making them a special case of a Petri net. The GNN frameworks draw comparison, furthermore, to *traditional* message passing techniques, presented next.

To aid further understanding, a brief overview of the GNN message passing methodology is presented. Though different extensions with respect to the original GNN framework exist, they all share the same principal idea^42,43,54^, expressed as follows. The forward pass consists of a *message passing* phase

3 $$\begin{aligned} m_{u,v}^{\tau +1} = \Psi (h_{u}^{\tau },h_{v}^{\tau },\omega _{u,v}) \end{aligned}$$

defined with respect to a learnable function $\Psi$, usually referred to as message function, over pairs of hidden states $h_{u}^{\tau }, h_{v}^{\tau } \in {\mathbb {R}}^{D}$ at iteration step $\tau$, and edge feature $\omega _{u,v}: E \rightarrow {\mathbb {R}}^{+}$, ${\mathbb {R}}^{+} = \{ x \in {\mathbb {R}} \mid x>0 \}$. The term *learnable* is used, as conventional, to refer to learning-based function approximation. Here, $u,v \in V$ correspond to nodes and $\{u,v\} \in E$ to edges of a traditional graph $G = (V,E)$, defined with respect to a vertex set *V* and edge set *E* (for details on objects and notation, see “Preliminaries”). The hidden states $h_{u}^{\tau }$, at each node $u \in V$, are *updated* (Eq. 5) by an *aggregated* message $m_{u}^{\tau +1} \in {\mathbb {R}}^{D}$ (Eq. 4), according to

4 $$\begin{aligned} m_{u}^{\tau +1}= & AGG_{v \in {\mathcal {N}}(u)} (m_{u,v}^{\tau +1}) \end{aligned}$$

5 $$\begin{aligned} h_{u}^{\tau +1}= & \Phi (h_{u}^{\tau },m_{u}^{\tau +1}) \end{aligned}$$

where the aggregation in Eq. (4) is determined by the neighbourhood ${\mathcal {N}}(u)$, $\forall u \in V$, and the permutation-invariant aggregation operator *AGG* (e.g., sum, max, mean, etc.; note that the sum operator discriminates more graph structures, while other aggregators are shown to be less expressive and suboptimal^53^). Update (Eq. 5) is defined with respect to a learnable function $\Phi$, usually referred to as an update function. The message passing phase runs for ${\hat{T}}$ time steps. In the *readout phase*, a feature vector ${\hat{y}} \in {\mathbb {R}}^{D}$ is computed for the entire object *G*, using a readout function *R*, according to

6 $$\begin{aligned} {\hat{y}} = R(\{h_{u}^{{\hat{T}}} : u \in V\}). \end{aligned}$$

In a *heterogeneous* GNN setting, the graph *G* is commonly partitioned into *b* homogeneous sub-graphs $\{ G^{(l)} = (V,E^{(l)}), l \in L, b = |L| \}$^56–60^, with aggregation performed in two steps, i.e., *sub-graph aggregation*, aggregating neighbourhood information of the same type,

7 $$\begin{aligned} m_{u}^{{\tau +1}^{(l)}} = AGG_{v \in {\mathcal {N}}(u)} (m_{u,v}^{{\tau +1}^{(l)}}) \end{aligned}$$

and *inter-relation aggregation*, aggregating relation-specific embeddings in a non-concurrent pairwise fashion, i.e.,

8 $$\begin{aligned} m_{u}^{\tau +1} = AGG_{l \in L} (m_{u}^{{\tau +1}^{(l)}}). \end{aligned}$$

In a *hypergraph* GNN, the approach taken is similar, except that (implicitly) *E* is not considered a set of node-pairs (for the definition of a hypergraph, see “Hypergraphs”). In practice, however, existing GNN frameworks^61,62^ still resort to pairwise node intuition (i.e., hyperedge groups with pairwise edges, or hyperegde groups with *k*-hop neighbours). The hyperedge is, ultimately, broken down into a family of central nodes *u* and relative neighbourhood node sets ${\mathcal {N}}(u)$, while being treated as homogeneous pairwise connected sub-graphs. Other concepts, such as hypergraph-based diffusion models, e.g.,^64–66^, represent a special case of continuos timed Petri nets^26–29^. Transformer-based models, e.g.,^69,71,72^, offer a high level of representation, however, they still disregard directionality in the definition of a relationship (i.e., they encode symmetry with respect to semantic conversion), which reduces them to a special case of a Petri net. Furthermore, they are extensively simplified by the Petri net formulation, which decreases the number of required self-attention parameters (see, “Proposed framework” and “Examples”). Ultimately, by incorporating the notion of a Petri net into the formal representation of a GNN model, a form of multimodal message passing is introduced, afforded by the concept of heterogeneous network flow^23^. The framework flattens relationship structure at the expense of losing explicit semantics, while preserving interpretation under reversible correspondence, facilitating computation. Such formulation extends scope of expression in traditional GNN frameworks, as presented next.

### Proposed framework

#### Multimodal message passing

Interaction in interconnected subsystems is often described as a form of evolution process, which under steady state conditions conforms to a network flow^8,9^ (i.e., random walk, belief propagation or message passing^21,22^). Unlike traditional algebraic flow, heterogeneous network flow^23^ captures concurrent coupled events by encoding both flow and mass conservation (see, “Heterogeneous network flow”), allowing flow derivation across different semantic domains. Multilayer and temporal networks are implicitly encoded (the former as the underlying network structure^16,17,23^, the latter as a time-expanded graph^73^, a multilayer special case; for a simplified illustration of multilayer and heterogeneous flow networks see, Fig. 2a,c). The framework is, furthermore, defined with respect to a Petri net^23–25^, a generalised hypergraph maintaining a form of multilayer concurrency (for a simplified illustration of a Petri net and HFN-Petri net transformation see, Fig. 2b-d; for hypergraph and Petri net correspondence see, “Hypergraphs”, “Petri nets”, and Fig. 1). The HFN-Petri net transformation enables a flattening of the layered relationship structure (typical of multimodal networked systems), at the expense of losing explicit semantics (normally attached to nodes). Given reversible correspondence, however, interpretation is always preserved and outputs can uniquely be transformed from one mathematical object to the other, facilitating computation^23^.

**Fig. 3:**
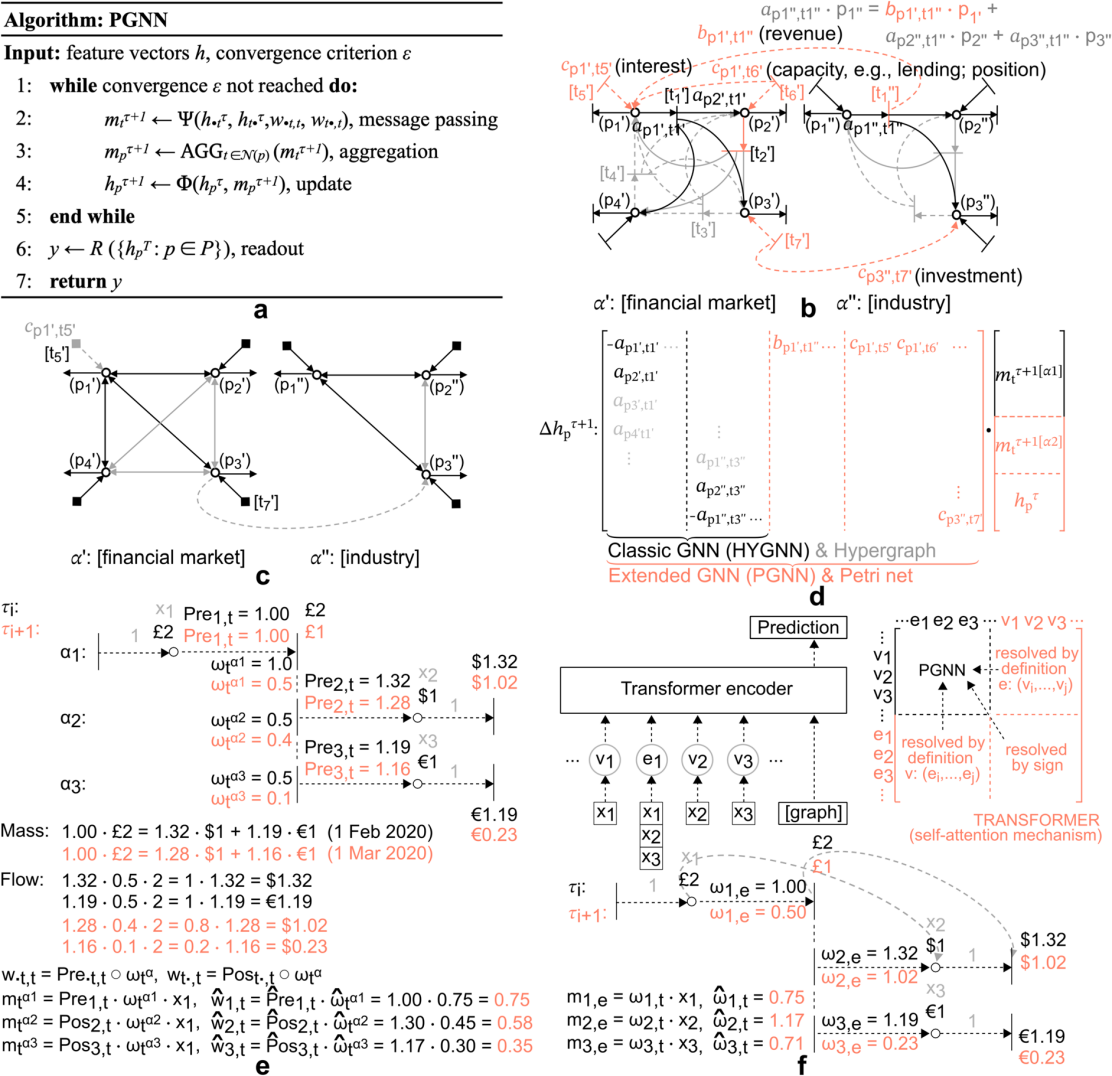
Illustrative example of PGNN algorithm, higher-order complex network, and corresponding GNN and PGNN context. **(a)** PGNN pseudocode algorithm. **(b)** Illustrative higher-order complex network, consisting of two hypergraphs ($\alpha ', \alpha ''$), forming layers of a multilayer network *M* ($V_{M} = \{ p'_{1},\ldots ,p''_{3} \}$, $E_{M} = \{ \{\{p'_{1}\},\{p'_{2},p'_{3},p'_{4}\}\},\ldots ,\{\{p''_{3}\},\{p''_{1},p''_{2}\}\} \}$). The structure encodes a simple synthetic financial problem, involving: financial market ($\alpha '$) and industrial sector ($\alpha ''$), exhibiting multilayer interaction (i.e., monetary ($\alpha '$) and product ($\alpha ''$) exchange). Note that interaction is: non-pairwise (e.g., four-body interaction of compound indices $p'_{1},\ldots , p'_{4}$), and concurrent (e.g., product exchange or transaction $t''_{1}$ in industrial sector, generates revenue for node $p'_{1}$ in financial market, acting as form of “tangible” node potential; the capacity of node $p'_{2}$ represents a type of “in-tangible” node potential for node $p'_{1}$, acting as form of relative position or borrowing power; both are extendable to multiple nodes). The figure depicts a PGNN interpretation of the higher-order node relationships, as multi-body concurrent interaction (note: $a_{p_{i},t_{j}}$ captures intra-layer node interactions; $b_{p_{i},t_{j}}$ captures coupling or exogenous force; $c_{p_{i},t_{j}}$ captures relative position; all correspond to *w* types; note process concurrency, i.e., all node states are derived simultaneously, across non-pairwise connected nodes). **(c)** GNN (HYGNN) interpretation of network in (b). Note pairwise node relationship and limits in expressing higher-order node interaction (i.e., a rectangle $p'_{1},\ldots , p'_{4}$ is expressed as six links $\{p'_{1}, p'_{2}\},\ldots , \{p'_{3}, p'_{4}\}$, not capturing four-body interaction, an entirely different case^40^). **(d)** Embedding incidence matrix for example in panel (b). **(e)** Illustrative example of mass conservation (based on daily, $\tau$, exchange rates^74^, $Pre_{p,t}^{\tau }$, $Pos_{p,t}^{\tau }$), and flow balance (based on daily, $\tau$, fund allocation, $\omega _{t}^{\alpha _{i},\tau }$), exhibiting multilayered hypergraph interaction across different semantic domains (i.e., currency layers, $\alpha _{i}$). **(f)** Panel (e) interpretation as TOKEN-GT^69^ (highlighting limits in representation). Furthermore, the number of parameters can significantly be reduced, based on simple rules of flow^8,9^ and network theory^44^, encoded by the PGNN^23–25^.

Heterogeneous network flow opens up a new direction for learning-based function approximation over networked structures, by offering a new type of mathematical object. In the context of a GNN, heterogeneous network flow introduces a form of *Multimodal Message Passing (MMP)*,

9 $$\begin{aligned} m_{t}^{\tau +1} = \Psi (h_{^{\bullet } t}^{\tau }, h_{t^{\bullet }}^{\tau }, w_{^{\bullet } t,t}, w_{t^{\bullet },t}) \end{aligned}$$

where $t \in T$ is a transition node of a Petri net $P_{N}$, and $^{\bullet } t = \{ p \in P: Pre_{p,t}> 0 \}$, $t^{\bullet } = \{ p \in P: Pos_{p,t}> 0 \}$, are sets of incoming and outgoing incident place nodes $p \in P$, respectively. Here, $h_{^{\bullet } t}^{\tau }$ is a vector of $|^{\bullet } t|$, and $h_{t^{\bullet }}^{\tau }$ a vector of $|t^{\bullet }|$ hidden states $h_{p}^{\tau } \in {\mathbb {R}}^{D}$ (note that, with reference to Eq. (3), Eq. (9) is defined with respect to a new mathematical object, see indices; for some additional context see, e.g., Fig. 3, illustrative example; note also that, for the sake of simplicity and with slight abuse of notation, node set *P* and *T* are assumed ordered, without loss of generality). A transition $t \in T$ allows any number of incident place nodes $p \in P$ to participate in its connections and is in part determined by them through incidence matrix *C* (see, “Petri nets”, “Heterogeneous network flow”, and Fig. 2f). The matrix encodes all weights (from places to transitions and transitions to places), represented here in vectors $w_{^{\bullet } t,t}, w_{t^{\bullet },t}$, where $w_{^{\bullet } t,t} = Pre \circ \omega _{t}$, and $w_{t^{\bullet },t} = Pos \circ \omega _{t}$. These weights correspond to both intra-layer flow rates (see, Fig. 2e, weights $\omega _{t}^{\alpha }$), as well as inter-layer conversion rates (see, Fig. 2e, *Pre* and *Pos* functions, where conservation and coupling constraints are both structurally encoded in the incidence matrix *C*, such that coupling can be interpreted as a form of exogenous flow balance, while conservation constraints correspond to a classic form of flow identity; for further reference see, e.g., “Heterogeneous network flow”, and Fig. 2c-f). The proposed object extends, therewith, traditional message passing expressed in Eq. (3), by encoding extended forms of node relationships, i.e., multi-body node interaction, process concurrency, and flow conversion or multimodality. Further details and formal proof are presented in the following paragraph.

#### Petri graph neural networks

The *Petri Graph Neural Network* (PGNN) offers an extended form of traditional GNN frameworks, by incorporating the notion of *multimodal message passing*, afforded by heterogeneous network flow^23^. The PGNN forward pass consists of an *MMP multimodal message passing* phase, in line with Eq. (9), followed by *MMP message aggregation*

10 $$\begin{aligned} m_{p}^{\tau +1} = AGG_{^{\bullet } p} (m_{^{\bullet } p}^{\tau +1}). \end{aligned}$$

Unlike Eq. (4), however, Eq. (10) is derived with respect to $^{\bullet } p = \{ t \in T: Pos_{p,t}> 0 \}$, a set of incoming transitions $t \in T$ incident with place nodes $p \in P$, where $m_{^{\bullet } p}^{\tau +1}$ is a vector of all $|^{\bullet } p|$ incoming messages $m_{t}^{\tau +1} \in {\mathbb {R}}^{D}$, encoding multi-body interaction with respect to *different semantic domains*. It therein offers simultaneous aggregation across: different semantic and concurrent coupled structures, i.e., *multimodal hypergraphs* (see, e.g., Fig. 3, arguments *b*), while capturing influence of incident potentials, with respect to adjacent nodes, i.e., *coupled exogenous forces* (see, e.g., Fig. 3, arguments *c*). The latter offers an important property, based on argument interpretation (i.e., $m_{t}^{\tau +1}$ as network flow, and $h_{p}^{\tau }$, $m_{p}^{\tau +1}$ as node potentials). Where Eq. (4), (7), (8), and their upgrades, capture direct pairwise node relationships^42,43,56–62^, or even some partial forms of higher-order node interaction^64–66^, relation Eq. (10) captures *higher-order multimodal node interdependency* $m_{t}^{\tau +1}$ and *relative position*, i.e., available capacity, $h_{p}^{\tau }$, $m_{p}^{\tau +1}$ (note that an object can be viewed as participating in a direct interaction in the form of mutual transaction or network flow, however, it can also act as an indirect “source” of potential; see, Fig. 3 and “Illustrative example”, for further context). In addition, the embedding matrix in a PGNN (see, Eq. (9)) corresponds to an incidence matrix, whereas in a GNN (see, Eq. (3)) it generally corresponds to adjacency (see, Fig. 3d for further reference; for the definition of incidence and adjacency, see “Graphs”; for the definition of a graph, hypergraph and Petri net, see “Preliminaries”).

The hidden states $h_{p}^{\tau }$ at each place $p \in P$ are *updated* according to

11 $$\begin{aligned} h_{p}^{\tau +1} = \Phi (h_{p}^{\tau },m_{p}^{\tau +1}) \end{aligned}$$

where, as previously noted, $m_{p}^{\tau +1}$ or Eq. (11) carries a higher level of intrinsic concurrent information, compared to Eq. (5). In the *readout phase* a feature vector ${\hat{y}}$ is computed for the entire object $P_{N}$, using a readout function *R* as

12 $$\begin{aligned} {\hat{y}} = R(\{h_{p}^{{\hat{T}}} : p \in P\}). \end{aligned}$$

For problems determined by a linear combination of feature vectors and Rectifier Linear Unit (ReLU)-based activation functions, relations Eq. (9)-(12) reduce to

13 $$\begin{aligned} {\hat{y}} = C h \end{aligned}$$

where *h* is a vector of node signals, while ${\hat{y}}$ is a vector of outcomes (this is afforded by an interpretation of node signals and outcomes as in- and outflows, respectively; see, e.g., Fig. 2c). Eq. (13) is a special case of a PGNN (see, Definition 1).

##### Definition 1

(Petri Graph Neural Network - PGNN) Let $H_{f}$ be a heterogeneous flow network and $P_{N}$ a Petri net, related by their corresponding HFN-Petri net transformation $T_{f}$. The Petri Graph Neural Network is an object of the form defined by relations Eq. (9)-(12).

Further expressive choice, relevant in adaptation to different computational settings, is introduced in Definition 2 and Remark 1. Lower bound properties, including proof of generalised performance of PGNN and MMP, are presented in Theorem 1 and Remark 2.

##### Definition 2

(Petri Net - Adjacency) Let $P_{N} = (P,T,Pre,Pos)$ be a Petri net. To an incidence matrix $C = Pos - Pre$ of $P_{N}$, we associate a multipartite adjacency matrix $A_{p} \in {\mathbb {R}}^{((|P|+|T|) \times (|P|+|T|))}$ of the form:

14 $$\begin{aligned} A_{p} = \left[ \begin{array}{cc} 0 & Pre \\ Pos^{T} & 0 \end{array} \right] \end{aligned}$$

such that

15 $$\begin{aligned} A_{p}' = A_{p}^{T} - A_{p} = \left[ \begin{array}{cc} 0 & C \\ -C^{T} & 0 \end{array} \right] \end{aligned}$$

16 $$\begin{aligned} A_{p}' z = 0. \end{aligned}$$

where $z = [x^{T} y^{T}]^{T}$, for any *P*-invariant vector *x*, and any *T*-invariant vector *y*.

##### Remark 1

Adjacency matrix $A_{p}$ (Eq. 14) can be derived from a form of incidence matrix *C* factorisation, following from the bipartite nature of a Petri net $P_{N}$ (note, places $p \in P$ and transitions $t \in T$ of a Petri net $P_{N}$, can be viewed as two different node types). The correspondence in Definition 2 is convenient in providing additional expressive choice, especially when, e.g., a Petri net or hypergraph incidence matrix is not a priori foreseen as a data structure (such as in, e.g., existing computational models or code), given that traditional GNNs generally learn the less expressive adjacency matrix.

##### Theorem 1

(Lower bound) The Graph Neural Network, expressed by Eq. (3)-(6), and its message passing extensions, are a special case of the Petri Graph Neural Network, Eq. (9)-(12).

##### Proof

The first part of the proof follows from an implicit property of higher-order complex structures, where pairwise node interaction (over graphs) is a special case of non-pairwise node relationships (over hypergraphs; see, “Preliminaries”). We begin by establishing a link between relations of node interaction, i.e., Eq. (3) and (9). A hypergraph (i.e., Petri net substructure) allows any number of nodes (i.e., places $p \in P$) to participate in its edges (i.e., transitions $t \in T$). For transitions with a single input $^{\bullet } t = \{ p_{u} \}$ and a single output $t^{\bullet } = \{ p_{v} \}$ (i.e., $h_{^{\bullet } t}^{\tau } = [h_{p_{u}}^{\tau }]$, $h_{t^{\bullet }}^{\tau } = [h_{p_{v}}^{\tau }]$, $w_{^{\bullet } t,t} = [w_{p_{u},t}]$, $w_{t^{\bullet },t} = [w_{p_{v},t}]$), the MMP relation in Eq. (9) reduces to

17 $$\begin{aligned} m_{t}^{\tau +1} = \Psi (h_{p_{u}}^{\tau }, h_{p_{v}}^{\tau }, w_{p_{u},t}, w_{p_{v},t}). \end{aligned}$$

Expressed as a graph $G = (V,E)$, where $p_{u}, p_{v} \in P$ correspond to nodes $u, v \in V$, respectively, and $t \in T$ to edges $\{u,v\} \in E$, such that $w_{p_{u},t} = w_{p_{v},t} = \omega _{u,v}$, it follows that

18 $$\begin{aligned} m_{u,v}^{\tau +1} = \Psi (h_{u}^{\tau }, h_{v}^{\tau }, \omega _{u,v}) \end{aligned}$$

demonstrating that Eq. (3) is a special case of Eq. (9). Given that Eq. (4)-(6) and Eq. (10)-(12) represent systems of equivalent functions, defined with respect to Eq. (3) and Eq. (9), respectively, it follows that Eq. (4)-(6) are a special case of Eq. (10)-(12), by extension. In addition, the heterogeneous GNN Eq. (7) is equivalent to Eq. (4) with respect to each layer $l \in L$, while Eq. (8) is equivalent to Eq. (5). Given pairwise node interaction (i.e., no *n* to 1 or 1 to *n* transitions $t \in T$), Eq. (7) corresponds to a form of pairwise intra-layer node relationship, while Eq. (8) corresponds to a form of pairwise inter-layer aggregation, both relating to a form of 1 to 1 transition $t \in T$, as expressed in Eq. (17). The same holds for hypergraph GNNs, which are ultimately broken down into hyperedge sub-graphs with pairwise node connections. Multi-body node interaction is expressed by definition in the proposed framework, i.e., Eq. (9), given that a Petri net $P_{N} = (P,T,Pre,Pos)$ represents a generalised hypergraph $H = (V,E_{H})$. Unlike a hypergraph *H*, the Petri net $P_{N}$ encodes both, n-body interaction (see, transition nodes $t \in T$), as well as multimodal node interaction (through relationships of mass conservation; see, e.g., “Hypergraphs”, “Petri nets”, i.e., the definition of hypergraphs $H_{G}$ and their node set *V*, as opposed to Petri nets $P_{N}$, their place set *P*, and *Pre* and *Pos* functions; see, Fig. 1, Fig. 2). On the other hand, a multilayer network $M = (V_{M},E_{M},V,L_{M})$, which can be utilised to encode multimodal node relationships, cannot encode multi-body node interaction, given formulation with respect to node-pairs (see, e.g., “Multilayer networks”, “Petri nets”, i.e., the definition of multilayer networks *M* and their edge set $E_{M}$, as opposed to Petri nets $P_{N}$ and transition set *T*; see, Fig. 2). A hypergraph $H_{G}$ is, hence, a special case of a Petri net $P_{N}$, such that $Pre = Pos = 1$, while a multilayer network *M* is a special case reducing the Petri net $P_{N}$ to pairwise connected transitions $t=\{p_{u},p_{v}\}$, i.e., $\{(u,\alpha ^{i}),(v,\alpha ^{j})\} \in E_{M}$. Hypergraphs $H_{G}$ and multilayer networks *M* are, furthermore, formally expressed as independent higher-order node dependencies^11–14^ (for further reference see, “Preliminaries”), while a Petri net $P_{N}$ encodes both types of higher-order node relationships^23,26–29^, as presented here, concluding the proof. $\square$

##### Remark 2

It is worth noting that the PGNN retains generality in the choice of aggregation Eq. (10), update Eq. (11), and readout functions Eq. (12); the difference lies in the *message passing phase* Eq. (9), which effectively is a form of network flow (here higher-order multimodal complex network flow, afforded by heterogeneous network flow^23^; see, e.g., Eq. (3), Eq. (9), and Fig. 3a). In the context of the Weisfeiler-Lehman graph isomorpihsm test^70^, this modification increases expressive power of the proposed framework in the following way. Information propagation in a graph (Fig. 1b) takes place with respect to node-pairs, e.g., $\{u_{i}, u_{j}\}, \{u_{i}, u_{k}\}, \{u_{j}, u_{k}\}$, aggregating information with respect to *adjacent nodes* (i.e., $m_{u_{k}}^{\tau +1} = AGG_{v \in {\mathcal {N}}(u_{k})} (m_{u_{i},u_{k}}^{\tau +1}$, $m_{u_{j},u_{k}}^{\tau +1}), m_{u,v}^{\tau +1} = \Psi (h_{u}^{\tau },h_{v}^{\tau },\omega _{u,v})$). In a hypergraph (Fig. 1c), this phase generalises to *n*-body connections, e.g., $e = \{u_{i},\ldots , u_{j}\}$, aggregating information with respect to incident hyperedges *e* (i.e., $m_{u_{k}}^{\tau +1} = AGG_{e \in {\mathcal {N}}(u_{k})} (m_{e}^{\tau +1})$), i.e., a *bipartite structure*. It is here worth noting that a hypergraph $H_{G} = (V,E_{H})$^15^ emerges as a special case of a Petri net $P_{N} = (P,T,Pre,Pos)$^24,25^ where, in structural terms, nodes $u \in V$ correspond to places $p \in P$, while hyperedges $e \in E_{H}$ correspond to transitions $t \in T$ (see, e.g., Fig. 1c,d), incident to all nodes in *e* with all possible combinations. The Petri net $P_{N}$ encodes, furthermore, relationships of multimodal node interaction, given compliance with laws of mass conservation. Unlike a hypergraph $H_{G}$, a Petri net $P_{N}$ is also a model of information flow^23,26–29^ (see, “Petri nets”, *P*- and *T*-invariants), where the message passing phase takes place with respect to *incident transitions* *t*, and *flow conversions* determined by *Pre* and *Pos* functions (i.e., $m_{p}^{\tau +1} = AGG_{t \in {\mathcal {N}}(p_{k})} (m_{t}^{\tau +1})$). A hypergraph $H_{G}$ is, hence, a special case of a Petri net $P_{N}$, such that $Pre = Pos = 1$, while a graph *G* is a special case reducing the Petri net $P_{N}$ to pairwise connected transitions $t=\{p_{u},p_{v}\}$. The same holds for a multilayer network $M = (V_{M},E_{M},V,L_{M})$^16,17^, given its definition with respect to node-pairs, i.e., $\{(u,\alpha ^{i}),(v,\alpha ^{j})\} \in E_{M}$.

The PGNN is, furthermore, a generalised form of the Tokenized Graph Transformer TOKEN-GT^69^, and the Hypergraph Message Passing algorithm HYPER-MP^67,68^, retaining *k-Weisfeiler-Lehman properties by extension*. TOKEN-GT is a generalised form of traditional transformer variants on graphs with graph-specific model formulations^69^. It encodes network components (e.g., nodes $u \in V$, hyperedges $e \in E_{H}$) as one object type, i.e., *tokens* $q_{u}, q_{e} \in Q$, subjecting them to a form of *self-attention mechanism*, i.e., embedding them in a complete (al-to-all) graph (for visualisation see, e.g., Fig. 3f). The transformation, however, eliminates directionality in the definition of a relationship (i.e., it encodes symmetry with respect to semantic conversion; e.g., $2H_{2}+O_{2} \rightarrow 2H_{2}O$, but also all other possible combinations thereof, such as $2H_{2}+2H_{2}O \rightarrow O_{2}$, which is clearly inconsistent with the physical meaning of the process). The network emerges as a special case of a Petri net, where tokens $q_{u} \in Q$ corresponds to places $p \in P$, while tokens $q_{e} \in Q$ correspond to transitions $t \in T$, incident to all nodes in $q_{e}$ with all possible combinations. Furthermore, the self-attention mechanism can be encoded in a more compact form with a Petri net formulation, where directionality of node-to-hyperdge connection is resolved by *sign* (i.e., *C* matrix formulation), while node-to-node and hyperedge-to-hyperedge connections are resolved by definition (i.e., as hyperedges and incident nodes, respectively; see, “Graphs”, “Hypergraphs”, “Petri nets”, for definitions; for some additional context and visualisation, see illustrative example in Fig. 3e,f). The PGNN offers, furthermore, an added level of representation learning through *Pre* and *Pos* functions (disregarded in TOKEN-GT, where *Pre* = *Pos* = 1, with focus placed on flow rate weights $\omega$; for additional context and visualisation, see illustrative example in Fig. 3e,f). The same holds for HYPER-MP^67,68^, which only offers a variation on the traditional message passing phase Eq. (3) (i.e., it adds information propagation and aggregation stages with respect to the hyperedge). The framework, however, disregards semantic conversion similar to other presented frameworks (assuming *Pre* = *Pos* = 1). It is also wort adding that, though TOKEN-GT implies departure from message passing algorithms, it *incorporates information propagation* mechanisms after all, given its self-attention stage (i.e., node relationships expressed as weighted graphs, can be encoded as network flows^8,9^). The framework extends, however, beyond traditional message passing algorithms, while executing in $O(n+m)$ time^69^. Overall, the findings presented here demonstrate that PGNNs have at least the same expressive power, meaning that their parameters can be tuned to replicate the predictions of other traditional GNNs. On the other hand, for datasets where higher-order node interactions are significant, PGNNs can afford better predictions.

Ultimately, the proposed framework *generalises message passing GNNs*, by extending their scope of expression (see, Fig. 1). Beyond hypergraph (multi-body) evolution, the proposed object encodes flow conversion (multimodal) and concurrent (parallel) interplay between different semantic domains. For problems not exhibiting this type of complexity, the object automatically reduces to its special case. Overall, the proposed PGNN framework offers: *structural transformation*, affording multimodal hypergraph flow derivation, extending beyond the state-of-the-art; *framework interpretation*, facilitating implementation and formal extension, including in traditional GNN settings as the PGNN special case; and *expressive power*, enhancing versatility, application potential and computational efficiency (within the context of concurrent systems and parallel computation^25,46^). The PGNN exhibits, furthermore, expressive power *as powerful as the k-WL* graph isomorphism test, while matching $O(n+m)$ time. The presented theoretical proof is, furthermore, corroborated by empirical evidence as well, where illustrative and real-world examples demonstrate superior performance of the proposed PGNN framework, as outlined next.

### Examples

#### Illustrative example: Higher-order multimodal complex interaction

The illustrative example is introduced to facilitate exposition of the proposed framework. It consists of a simple synthetic financial problem, comprising two sets of random variables (Fig. 3b), i.e.: the financial market ($\alpha '$) and industrial sector ($\alpha ''$), exhibiting multilayer interaction (i.e., monetary ($\alpha '$) and product ($\alpha ''$) exchange). In traditional GNN terms (Fig. 3c), node interaction would be encoded in network structure as pairwise node relationships, capturing some semantics implicitly, given learnable functions. These interactions, however, would not account for event concurrency and multimodal incidence (see, Fig. 3b,c), expressing only a share of direct pairwise node relationships. Multimodal incidence, a form of multi-body interaction with respect to different semantic domains (see, Fig. 3d, black and grey terms), encodes a form of *coupling* or exogenous force within a multilayer context (see, Fig. 3b,d, argument *b*), as well as *relative position*, i.e., a type of indirect source of potential or available capacity (see, Fig. 3b,d, argument *c*). In the context of the presented example, this exogenous inflow can correspond to, e.g., revenue generated by the industrial sector (see, e.g., Fig. 3b, transition $t_{1}'$), transferred into the financial market (e.g., into place node $p_{1}'$, with interest or coupling $b_{p1',t1'}$), increasing the financial *potential* (e.g., purchase or lending power of sink node $p_{1}'$ with respect to, e.g., nodes $p_{2}'$, $p_{3}'$, $p_{4}'$). At the same time, the financial potential (see, e.g., transition $t_{6}'$) can influence the movement in other nodes as well (through, e.g., its influence or relative position $c_{p1',t6'}$), which can be interpreted in the context of exogenous flow (i.e, feature vectors *h* become a form of message or inflow as well; see, Fig. 3d).

**Fig. 4:**
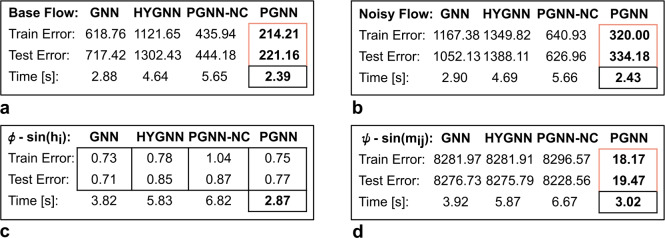
Illustrative example scenarios. Training and test error, with computational time, for: **(a)** baseline, **(b)** noisy, **(c)** “flattened”, and **(d)** nonlinear message passing, with respect to learning paradigm (GNN: message passing graph neural network; HYGNN: hypergraph neural network; PGNN-NC: Petri graph neural network disregarding notion of concurrency; PGNN: proposed Petri graph neural network framework).

**Fig. 5:**
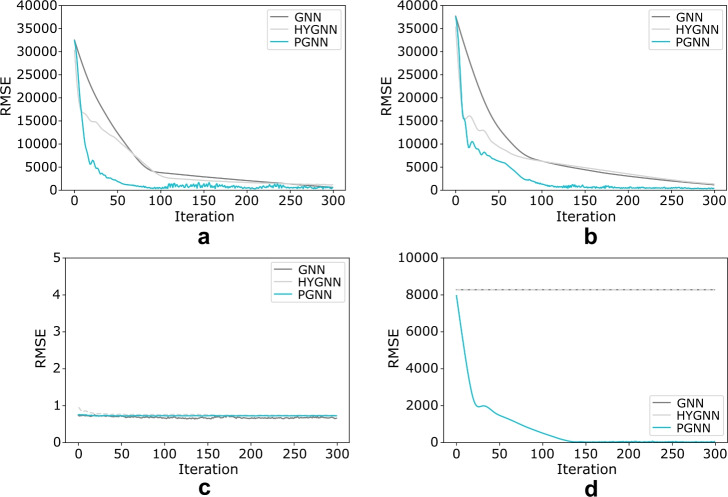
Illustrative example learning curve. Learning curve root-mean-square error (RMSE), for: **(a)** baseline, **(b)** noisy, **(c)** “flattened”, and **(d)** nonlinear message passing, with respect to learning paradigm (GNN: message passing graph neural network; HYGNN: hypergraph neural network; PGNN-NC: Petri graph neural network disregarding notion of concurrency; PGNN: proposed Petri graph neural network framework).

To provide some further context, a couple of illustrative examples are presented. A simple synthetic dataset is derived, involving 100 samples, produced based on Eq. (13) and Fig. 3b (i.e., Eq. (13), a simple special case scenario, as elaborated in “Petri graph neural networks”). The dataset consists of randomly generated node signals *h* (uniformly distributed over a sampling range $\{1,\ldots ,1000\}$), and independent realisations of labelled outcomes ${\hat{y}}$ (obtained from Eq. (13), with respect to a synthetic ground truth incidence matrix *C* corresponding to Fig. 3b, consisting of fixed arbitrary weights uniformly distributed over a range $\{0,\ldots ,10\}$; in the noisy scenario, node signals *h* and labelled outcomes ${\hat{y}}$ are perturbed by additive uniformly distributed noise). Assessed models correspond to: GNN (i.e., MPNN, Eq. (3)-(6))^42,43^, HYGNN (i.e., the “hypergraph” MPNN with pairwise node interaction and sequential updates, Eq. (3)-(8))^61,62^, PGNN-NC (i.e., a HYGNN with added coupling, see Fig. 3d columns *b*, disregarding concurrency), and the proposed PGNN (i.e., the higher-order complex network with multimodal concurrent coupling, Eq. (9)-(12)). In the PGNN-NC model, the update function Eq. (8) extends to $m_{u}^{\tau +1} = AGG_{l \in L} (m_{u}^{{\tau +1}^{(l)}}, \omega _{u}^{l})$ (see, Fig. 3d, columns *b*). The models contain no additional architecture; the example only serves to provide comparative analysis and illustrative presentation of different learning paradigms. Each model is trained over a maximum of 300 epochs (iterations), using the Adam^75^ optimiser (and a uniform training to test split of 70% to 30%). The PGNN learns $m + n \cdot m$ parameters (i.e., flow rate weights $\omega$, and flow conversion functions *Pre*, *Pos*, respectively), with respect to the underlying heterogeneous flow network structure^23^, as opposed to, e.g., $(n+m) \cdot (n+m)$ parameters (i.e., flow rate weights $\omega$) by TOKEN-GT, with respect to transformers on graphs^69^ (see, e.g., Fig. 3e,f).

The first case (Fig. 4a, 5a), corresponds to the simple baseline scenario. Here PGNN outperforms GNN and HYGNN by three times, and PGNN-NC by two times, in both training and test error. If the data is perturbed by additive (uniformly distributed) zero-mean noise (Fig. 4b, 5b), the proposed PGNN only produces a slight error increase, whereas the GNN and HYGNN errors almost double, with PGNN outperforming both by almost four times, and PGNN-NC by two times (the signals are left unprocessed, to test different learning environments). It is worth stressing that, as the models share the same function approximation mechanisms, the true limitation for GNN-based frameworks lies in identifying a structure beyond their scope of expression. If the node signals are “flattened” (Fig. 4c, 5c), the PGNN and GNN-based frameworks perform equally well (note that both conduct a form of learning-based function approximation, hence errors should be viewed within a margin). This further demonstrates the PGNN lower bound property (i.e., at least as good a result as GNN-based frameworks; see, Theorem 1). Unlike GNN, however, PGNN captures the structure of the underlying relationships more closely. This is especially evident in the last scenario, where nonlinear message passing (Fig. 4d, 5d) is imposed. While the proposed PGNN captures the underlying relationship structure successfully, the GNN and HYGNN, as well as PGNN-NC, start exhibiting a form of “divergent” behaviour.

The proposed PGNN demonstrates, furthermore, superior computational power as well, achieving less than half the HYGNN computational time and about less than 20% of the GNN time, with better scaling (note that the number of computed parameters in a PGNN is at least two times higher compared to a GNN or HYGNN, given concurrent relationships between place and transition nodes, acting as a source of exogenous force - see, e.g., Fig. 3b,d for context; computational power, however, is attributable to the parallelisation afforded by the Petri net object^25,46^). It is also worth adding that interpretations used in the formulation of the PGNN model are implemented in the composition of the competing GNN models as well, facilitating their performance. The PGNN, however, still outperforms the state-of-the-art due to higher expressive power, identifying structure beyond traditional graph, hypergraph or multilayer context, improving results and facilitating the overall learning process. It is also worth adding that any type of ablation analysis would still result in a better performance of the proposed Petri net framework, given generality of the underlying structure (see, Theorem 1 and proof).

**Fig. 6:**
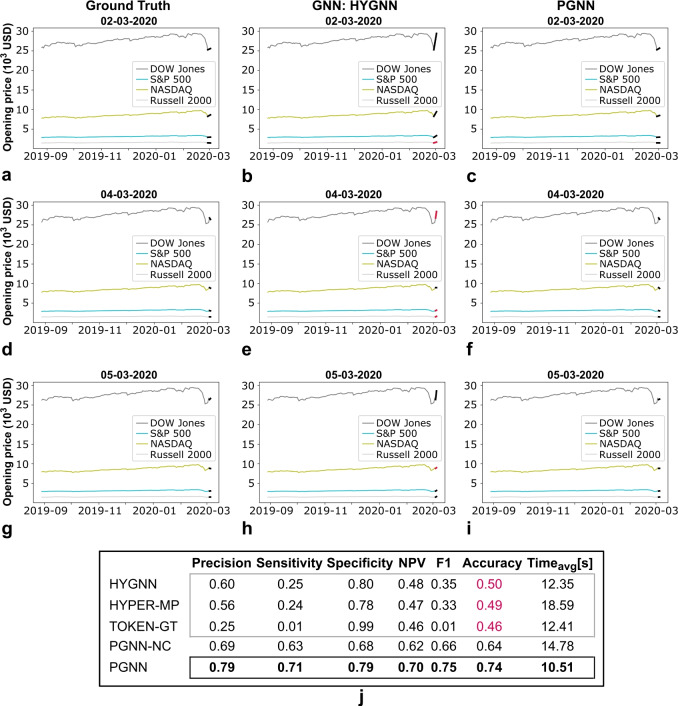
Example of opening price predictions for four major stock market composite indices (i.e., DOW Jones, NASDAQ, S&P 500, Russell 2000), based on traditional GNN and the proposed PGNN framework. Predictions for dates: **(a)**-**(c)** 02–03-2020, **(d)**-**(f)**, 04-03-2020, and **(g)**-**(i)** 05-03-2020 (note onset of the global pandemic). Plots (a), (d), (g) correspond to the ground truth, (b), (e), (h) to the GNN-based model, and (c), (f), (i) to the PGNN model. **(j)** Performance metrics for the full year (2020), demonstrating superior performance of the proposed PGNN framework. Note that the GNN-based model achieves 50% accuracy, pointing to randomly generated results. The upgraded model with added coupling (disregarding the notion of concurrency, PGNN-NC) produces improved results, however, still lacks the context of relative position and incidence, captured by the PGNN. (Performance metrics computed as: $Precision = TP/(TP+FP)$, i.e., positive predictive value; $Sensitivity = TP/(TP+FN)$, i.e., recall; $Specificity = TN/(TN+FP)$; $NPV = TN/(TN+FN)$, i.e., negative predictive value; $F1 = 2 \cdot Precision \cdot Sensitivity / (Precision + Sensitivity)$; and $Accuracy = (TP + TN)/(TP+TN+FP+FN)$; where *TP* corresponds to true positives (or rise), *TN* to true negatives (or fall), *FP* to false positives (or falsely predicted rise), and *FN* to false negatives (or falsely predicted fall). $Time_{avg}$ corresponds to the average computation (training) time, per diurnal prediction).

**Fig. 7:**
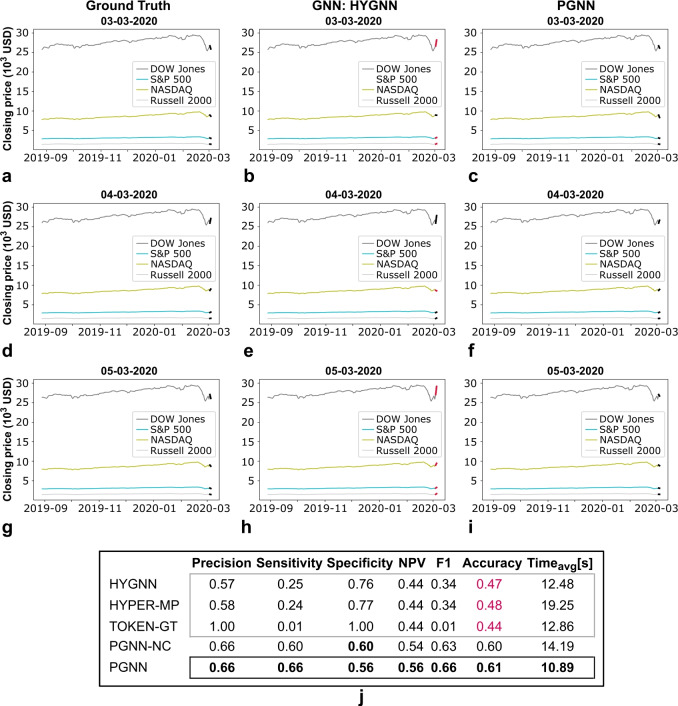
Example of closing price predictions for four major stock market composite indices (i.e., DOW Jones, NASDAQ, S&P 500, Russell 2000), based on traditional GNN and the proposed PGNN framework. Predictions for dates: **(a)**-**(c)** 03-03-2020, **(d)**-**(f)**, 04-03-2020, and **(g)**-**(i)** 05-03-2020 (note onset of the global pandemic). Plots (**a**), (**d**), (**g**) correspond to the ground truth, (**b**), (**e**), (**h**) to the GNN-based model, and (**c**), (**f**), (**i**) to the PGNN model. **(j)** Performance metrics for the full year (2020), demonstrating superior performance of the proposed PGNN framework. Note that the GNN-based model achieves 47% accuracy, pointing to randomly generated results. The upgraded model with added coupling (disregarding the notion of concurrency, PGNN-NC) produces improved results. Though the model still lacks the context of relative position, in this case such impact is less pronounced. The context of coupling, nonetheless, still remains an important feature, pointing to the relevance of the expressive power offered by the proposed PGNN framework. (Performance metrics computed as before.).

#### Real-world example: Stock market price prediction

To demonstrate performance of the proposed PGNN framework in a real-world setting, an example of a stock market price prediction model is presented. The model captures movement of four major stock market composite indices (i.e., DOW Jones, NASDAQ, S&P 500, Russell 2000)^76^, for the time period 2012-2020. The problem consists of predicting daily closing and opening price movements for each index in 2020, based on historical daily records. The signals relate to a highly stochastic process, with target year records additionally exacerbated by the onset of the global pandemic. To aid predictions, the data is complemented by historical records of commodity futures prices (i.e., gold)^76^. These provide a form of multilayer intuition, given movement on a semantically different plane (i.e., futures contracts, operating on a different set of mechanisms - commodity futures correspond to locked-in prices, reflecting current beliefs with respect to a futures contract, i.e., remuneration on a predefined future date^76^). The function approximation models are formulated as in the “Illustrative example”, except that inputs are concatenated signals $[h_{O}^{\tau -1} \Vert h_{C}^{\tau -1} \Vert h_{c}^{\tau -1} \Vert z^{\tau -1}]$ (where $\Vert$ denotes concatenation), corresponding to historical opening $h_{O}^{\tau -1}$ and closing prices $h_{C}^{\tau -1}$, commodity futures $h_{c}^{\tau -1}$, and opening/closing price trends $z^{\tau -1} = z(h_{O}^{\tau -1},h_{C}^{\tau -1})$. In the PGNN model, signals are expanded by positional opening/closing price and commodity futures data (i.e., coupling and relative position - see, “Petri graph neural networks” and Fig. 3). All signals are standardised, on a rolling basis. Diurnal trends are predicted for each day of 2020, based on 8-year historical records of opening and closing prices^76^. The cut-off limit $\varepsilon$ for trend classification $|z^{\tau }|> \varepsilon$ (i.e., rise or fall) is set to $\varepsilon = 0.1\%$ for DOW Jones and NASDAQ, and $\varepsilon = 0.5\%$ for S&P 500 and Russell 2000 (due to slow movement, i.e., low second moment; it is wort adding that only $2\%$ of predictions, i.e., a one week equivalent, fell within the cut-off range $|z^{\tau }| \le \varepsilon$, i.e., not predicting either of the respective trends/classes, for the entire range of diurnal predictions over a full year). Given only one futures commodity (as second semantic layer), and a complete (i.e., one-to-one) graph, the GNN and HYGNN models (see, formulation in “Illustrative example”) effectively correspond to one in this case. It is worth adding that the example serves to demonstrate the extent of expressive power offered by the proposed PGNN framework (i.e., comparative analysis with respect to the state-of-the-art), rather than the formulation of a detailed financial model. Implementation can be easily extended to other more detailed financial models, or other application domains.

Based on the obtained results (Fig. 6), the proposed PGNN improves predictive power of the financial model to 73.5% accuracy, from a 50.4% achieved by traditional GNN frameworks. Even if the GNN-based model is upgraded, such as to include coupling (i.e., Fig. 3d, columns *b*) in the form of a PGNN-NC (i.e., incorporating conversion, but disregarding the hypergraph, i.e., *n*-body interaction), the model still lacks expressive power to encode relative position (i.e., Fig. 3d, columns *c*) and concurrency (i.e., Fig. 3d, grey terms, all columns), with accuracy increasing to 64.2% only. PGNN outperforms the respective models across both positive (PGNN 70.7% vs. upgraded GNN 63.2%) and negative (PGNN 78.6% vs. upgraded GNN 67.7%) opening price movements (i.e., rise and fall; Fig. 6a-i), with an overall improvement in performance metrics (Fig. 6j). The same holds for closing prices as well, where the PGNN results in 60.7% accuracy, as opposed to 47.2% obtained by traditional GNNs (i.e., 59.9% if upgraded; note that in this case, relative position stops playing a significant role in the prediction of the outcome; coupling, however, is still prevalent and requires consideration, as derived from the PGNN-NC and PGNN results; see, Fig. 7). It is here also worth adding that PGNN produces results at 75% of the GNN time. In addition, given a highly volatile process, a successful prediction of true positives above 50% points to a model that captures the underlying mechanisms or markers driving the price changes to a certain extent. This is based solely on *the underlying relationship paradigm* (i.e., the models contain *no additional architecture*, just the corresponding mathematical object/framework), where the focus of this example is simply to provide a comparative analysis (rather than the formulation of a detailed financial model). The main goal is to demonstrate that the proposed framework enables capturing financial markers beyond traditional GNN intuition, with respect to the same baseline, due to more expressive power and added interpretation. As such, it can serve as an additional mathematical tool in the formulation of, e.g., a more detailed financial model, or other learning-based function approximation applications, which is an independent problem, outside the scope of this manuscript.

## Conclusion

We propose the Petri Graph Neural Network, i.e., PGNN, as a novel learning framework that extends traditional GNNs by enabling higher-order, multimodal, and concurrent information propagation across semantic domains. Unlike hypergraphs, which cannot enforce semantic conversion, or multilayer networks, which cannot capture multi-body dependencies, the PGNN leverages Petri nets to model complex interactions through heterogeneous network flow. This allows both multi-body and multi-semantic relationships to be represented simultaneously and reversibly, preserving interpretability and enabling efficient computation. By generalising message passing beyond existing GNN paradigms, PGNN offers increased expressiveness and applicability to real-world systems characterised by higher-order structures, such as brain networks, protein interactions, genetic systems, and socio-economic networks. Our theoretical analysis and empirical results (e.g., in stock market prediction) validate its effectiveness. Finally, PGNN opens new avenues for research in hypergraph-based learning, transformer-based algorithms, geometric understanding of higher-order relationships, and safe deployment in critical domains requiring interpretable models, which are areas of interest for future work.

## Data Availability

Datasets used in this manuscript are available through public repositories: https://uk.finance.yahoo.com/.
